# Efficacy of an Educational Health Intervention for Oral Cancer Aimed at Dentists and Dental Students

**DOI:** 10.1007/s13187-025-02644-9

**Published:** 2025-06-18

**Authors:** Gloria Cristina Aranzazu-Moya, Gloria Jeanethe Álvarez Gómez, Eliana Elisa Muñoz López, Yenny M. García Tarazona, Gloria Cristina Moreno Abello, Sandra Milena Espitia Nieto, Dora Eugenia Ordoñez Daza, Farley Piedad Aguinaga Rodríguez, Leonor Victoria González Pérez, Claudia Patricia Peña Vega, Zoila Carbonell Muñoz, Sandra Juliana Rueda Velásquez

**Affiliations:** 1 School of Dentistry, Universidad Santo Tomás , Bucaramanga, Santander, Colombia; 2 School of Dentistry, Universidad de Antioquia, Medellín, Antioquia Colombia; 3 School of Dentistry, Universidad Autónoma de Manizales, Manizales, Caldas, Colombia; 4 School of Dentistry, Universidad El Bosque , Bogotá, DC Colombia; 5 School of Dentistry, Pontificia Universidad Javeriana, Bogotá, DC Colombia; 6 School of Dentistry, Universidad del Norte, Barranquilla, Colombia; 7 School of Dentistry, Universidad del Valle, Cali, Valle del Cauca Colombia; 8 School of Dentistry, Institución Universitaria Vision de las Americas , Medellín, Antioquia Colombia; 9 School of Dentistry, Universidad Nacional de Colombia, Bogotá, DC Colombia; 10 School of Dentistry, Universidad de Cartagena, Cartagena de Indias Bolivar, DT y C Colombia

**Keywords:** Knowledge, Attitude, Professional practice, Oral cancer, Dentist, Dental Students, Online education

## Abstract

The objective of this study was to examine the efficacy of massive open online courses (MOOCs) for implementing standardized training and teaching with respect to oral cancer among dental students and dentists. In a multicenter interventional randomized controlled trial, a total of 660 Colombian dental students and dentists were randomly assigned to one of three groups between September 2022 and August 2023 by means of the random number table method at ten different institutions involved in dental education. With a loss to follow-up rate of 25%, Group 1 consisted of 107 cases, Group 2 consisted of 177 cases, and the control group consisted of 164 cases. The control group did not receive training, whereas Group 1 received a leaflet, and Group 2 participated in a MOOC-based learning approach. The assessment results of the knowledge, attitudes, and practice scores were observed and compared among the three groups of participants before and 2 months after the intervention. Compared with the control group, Group 2 presented significantly higher scores for KAP (*P* < 0.05). The group also showed greater improvements in terms of knowledge, attitudes, and practices (*P* < 0.05) than did the control group. Additionally, the KAP scores of the students in Group 2 were significantly higher than those of the dentists. Group 2 also demonstrated a substantially greater size effect in KAP. The implementation of MOOC teaching was shown to positively affect standardized training and teaching related to oral cancer, thereby enhancing academic performance in this area.

Trial registration: ISRCTN48708543.

## Introduction

In 2020, cancers of the lip, oral cavity, and oropharynx were the sixteenth (13 th) most common type of cancer worldwide and the most common malignancies of the oral cavity [1]. By 2022, cancers of the lip and oral cavity ranked 16 th in incidence, and those of the oropharynx ranked 24 th [2]. These rates have decreased in some countries as tobacco consumption has decreased; however, secondary to human papillomavirus infection, some countries have experienced an increase in the incidence of oropharyngeal cancer among individuals under 60 years of age. In this sense, given that records are maintained differently across countries, it is difficult to prove this statement, although the Colombian statistics suggest similar patterns [1], as reported in the population-based cancer registry in Cali, Colombia, which showed stable figures for HPV-related cancers and a reduction in cancers related to habits such as smoking and alcohol consumption [3].

The incidence rates reported by the National Institute of Health (NIH) in the USA for 2022 show an increase in the incidence of cancer in the oral cavity and pharynx in men, with an average annual percentage change (AAPC) of 0.2, and in women, with an AAPC of 0.7 between 2014 and 2018. On the other hand, mortality from the same cancer in the oral cavity and pharynx in men decreased with an AAPC of − 0.7 between 2015 and 2019; however, the same did not occur in women, for whom the mortality for the same area in the AAPC was 0.3 [2, 3]. The incidence rates in Latin America are not very high, but in Colombia, from 2018 to 2022, the mortality of lip, mouth, and pharyngeal cancer ranged from 15 (2.6%) to 19 (3.3%) among men and 15 (2.1%) to 20 (2.9%) among women, ranking these cancer types among the 10 leading causes of death from cancer, indicating an increase in the number of deaths from this type of cancer [4, 5].

In the USA, a 10-year survival rate of 87% has been reported for oropharyngeal cancer, followed by oropharyngeal cavity cancer (69%), larynx cancer (67%), and oropharynx-P16 + cancer (56%) [6]. The 5-year survival rate in Colombia does not exceed 54% [7, 8]. This has been related to an increase in the time elapsed between the first symptom and the time of diagnosis, which results in an advanced stage and increased mortality [9].

In this sense, late detection leads to a higher percentage of patients with stage IV cancer, as revealed by previous studies [10], which leads to a poor prognosis [11].

To increase early diagnosis and reduce the time between the appearance of symptoms and the definitive diagnosis, it is necessary to educate patients about self-examination, the most relevant risk factors and timely consultation, not only to patients but also to general and specialist dentists and students of dentistry, who have the opportunity to perform a thorough examination of the oral cavity and oropharynx and, therefore, a timely diagnosis and referral [12].

Thus, the knowledge, attitudes, and practices of oral health professionals with respect to oral cancer have been evaluated in many studies, and all of them show a need to implement continuing educational strategies [13]. Therefore, the aim of this study was to evaluate the effectiveness of an educational strategy such as massive online open courses (MOOCs) with respect to the knowledge, attitudes, and practices of dentists and dental students in Colombia.

## Methods

In a controlled clinical trial, 660 participants were randomized, with 330 dental students and 330 dentists belonging to 10 dental schools of Colombian universities.

The sample size calculation was performed on the basis of the results of the intervention performed by Jeihooni and collaborators, who reported scores of 6.8 SD3.5 for the intervention group and 3.1 SD7.1 for the control group. Considering a power of 80% and a two-sided alpha of 5%, 22 subjects were assigned to each group at each participating institution. A total of 66 subjects from each of the 10 participating institutions were included.

The participants were randomized to one of three intervention groups: the brochure intervention group, the MOOC intervention group, and the control group without intervention.

Dentistry students who passed the oral pathology course and had started their clinical practice and dentists who were generalists or specialists were included. Dentists specializing in oral pathology, stomatology, oral medicine, and oral and maxillofacial surgery were excluded.

Two educational materials based on updated literature were designed, generating a six-body brochure and a MOOC with four sections (e.g., (1) epidemiology, statistics, and risk factors; (2) clinical considerations and potentially malignant disorders (PMD); (3) diagnostic strategies early, biopsy; and (4) risk factor management), texts, videos, cases, and self-evaluations for each section and a final evaluation. The MOOC was designed for 48 h of continuing education, and a certificate was generated.

To enhance participant motivation and ensure a high completion rate in this extended course, two key strategies were implemented:

- Structured progression with incremental goals: Each section of the course was designed to include concise, interactive learning activities (e.g., quizzes, reflections, or short assignments). Participants were required to complete these activities to unlock access to subsequent sections. This approach created a sense of accomplishment, maintained engagement, and encouraged steady progress through manageable milestones.
- Certification as a motivational incentive:

A certificate was awarded exclusively to participants who completed the entire course. This credential was framed not only as a professional asset but also as a tangible reward for their commitment. Regular reminders about the certification’s value such as its relevance to career development or skill recognition were communicated to reinforce its importance and sustain motivation over time.

These strategies aimed to balance immediate gratification (via section completion) with a long-term goal (certification), addressing both intrinsic and extrinsic motivational drivers.

The contents of the materials used were based on recent publications and were designed and evaluated by 10 specialized professionals in areas such as oral pathology, oral medicine, surgery, and stomatology.

The effects of the interventions were evaluated before and 2 months after the interventions via the CAP CaO-D questionnaire of knowledge, attitudes, and practices in oral cancer directed to dentists. This questionnaire was validated with 18 questions of knowledge, 17 questions of attitudes, and 17 questions of practices, with internal consistencies of 0.700, 0.856 and 0.840, respectively, and 0.878 for the complete questionnaire.

**Fig. 1:**
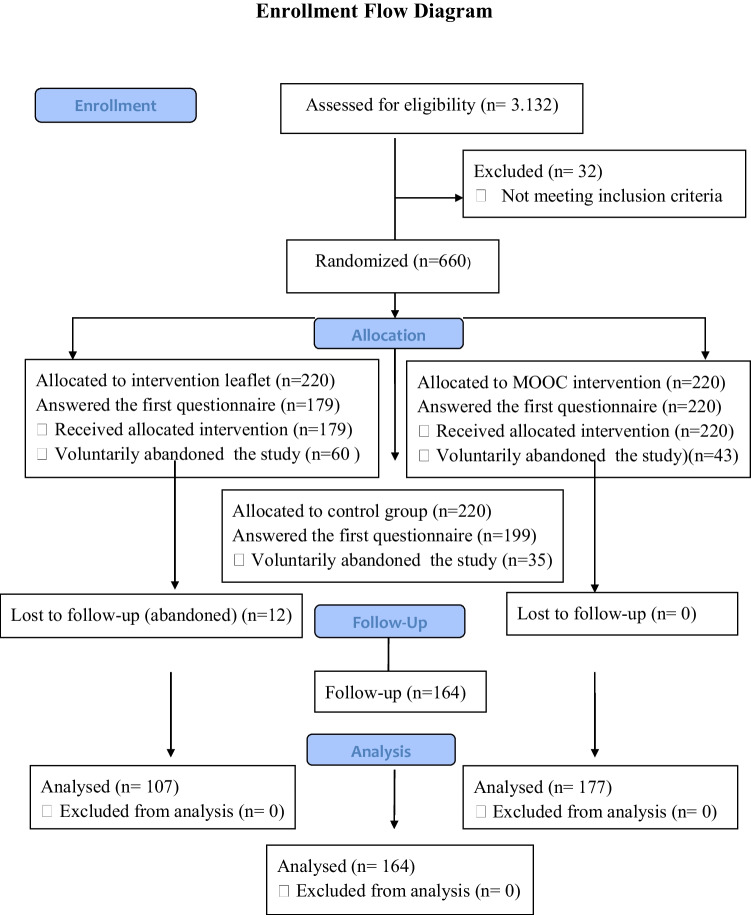
Enrollment flow diagram

The participants signed an informed consent form, and their personal information was anonymized, respecting their privacy and autonomy. Those randomized who rejected their participation were randomly replaced with the lists of each institution. A total of 660 participants were randomized, 25% left the study, and 448 participants finished the study; 107 received the brochure intervention, 177 received the MOOC intervention, and 164 participated in the control group (Fig. 1).

The project was registered on the trial registration platform with the number ISRCTN48708543 and was approved by the ethics, bioethics, and scientific integrity committee of the Universidad Santo Tomás with codes 0161–2021.

## Results

Initially, 660 participants who were randomized to the educational intervention and control groups were recruited. Of these, 598 answered the first questionnaire, with a nonresponse percentage of 9.4%. Finally, 448 people, including 133 men and 315 women, completed the study, with a loss to follow-up rate of 25%.

The knowledge, attitudes, and practices scores were calculated for the three randomized groups where a greater positive change was obtained in the three dimensions for the MOOC intervention. Similarly, higher scores were observed for students than for dentists (Table 1).

**Table 1:** Dental student and dentist KAP scores before and after the interventions

	Dental students Me (IQR)		Dentist Me (IQR)	
	Score before	Score after	Score before	Score after
Group 0	67 (57–74)	70 (61–75)	64 (54–69)	66 (55–74)
Group 1	71 (64–77)	77 (69–85)	62.5(55–69)	67.5(61–77)
Group 2	65 (51–72)	87.5 (80–92)	65 (57–72)	83 (77–89)

Group 0, without intervention; Group 1, intervention leaflet; Group 2, intervention MOOC

*Me*, median; *IQR*, interquartile range

According to the randomized group, total scores and knowledge, attitudes and practices were established, where no statistically significant differences were identified in the three groups in terms of the total score before the interventions. After the interventions, differences were evident in the three dimensions and in the total score of the questionnaire (Table 2).

**Table 2:** KAP scores and size effect by dimension between intervention groups

	Group 0 *n* = 164 Me (IQR)	Group 1 *n* = 107 Me (IQR)	Group 2 *n* = 177 Me (IQR)	*P* value *
Knowledge before	9 (8–10)	10 (8–10)	8 (7–10)	**0.0004**
Knowledge after	9 (8–10.5)	10 (9–11)	14 (12–15)	**0.0001**
**Size effect**	0.008 CI (0.11–0.09)¥	0.127 CI (0.25–0.006)§	**0.779 CI (0.81**–0.74)**	
Attitude before	19 (14–23)	19 (16–24)	18 (15–24)	0.318
Attitude after	20 (15–25)	24 (18–28)	32 (28–34)	**0.0001**
**Size effect**	0.055 CI (0.16–0.05)¥	0.252 CI (0.36–0.12)§	**0.683 CI (0.72**–**0.62)ª **	
Practice before	36 (30–40)	38 (33–41)	37 (31–42)	0.148
Practice after	38 (32.5–43)	40 (36–43)	42 (37–44)	**0.0001**
**Size effect**	0.150 CI (0.25–0.042)§	0.192 CI (0.31–0.06)§	**0.354 CI (0.43**–**0.26)ª**	
Score before	64.5 (54.5–72)	65 (57–74)	65 (55–72)	0.2789
Score after	68 (57.5–75)	73 (64–81)	86 (78–91)	**0.0001**
**Size effect**	0.121 CI (0.22–0.013)§	0.268 CI (0.38–0.14)§	**0.670 CI (0.71**–**0.61)ª**	

Group 0, without intervention; Group 1, intervention leaflet; Group 2, intervention MOOC

*Me*, mediate; *IQR*, interquartile range; *P = Kruskal‒Wallis; size effect = biserial R; ¥<0.1 Absent effect; §0.1-0.29 Small effect; ª >0.3-0.69 Large effect; **>/=0.7 Very large

When evaluating the effect size of the interventions, we found a small and absent effect in the group that received no educational intervention, a small effect size in the group treated with the brochure, a large effect for the total KAP and for the dimensions of attitudes and practices, and a very large effect on knowledge in the group with MOOC as the intervention (Table 2).

## Discussion

In studies that evaluate knowledge, attitudes, and practices in oral cancer patients, the conclusion is that it is necessary to implement strategies that improve these dimensions of action among health professionals, including both doctors and dentists [14]. This is the only way in which it is possible to reverse the trends with respect to the late diagnosis of the disease in stages III and IV and influence the trend so that it favors early diagnosis and improvements in the 5-year survival figures, therefore reducing the economic impact on health systems and the personal impact on patients and their families. For this reason, in this study, we evaluated an educational strategy designed to overcome barriers to access of continuing education, time management, and self-management [15–17].

It is important to establish that educational strategies always generate changes in knowledge and attitudes [18]. However, the MOOC designed in this study managed to better impact the knowledge, attitudes, and practices of the participants, resulting in a significant effect size. Given that the evidence indicates that 50% of the strategies do not include potentially malignant disorders or risk factors such as human papillomavirus or chronic traumatic injuries [14], the contents of the MOOC included these topics according to the level of evidence and current concepts. In this sense, this study also responds to recent publications, such as that reported by Martínez-Ramírez and collaborators, who reported that 100% of health professionals believe that online education is necessary, in addition to considering that there is a shortage of continuing education on the subject [15].

MOOCs facilitate access to information; they can be entered at any time and provide access to the internet, facilitate remote interaction, and allow the specialist to retake and review the material as many times as necessary. This facilitates self-management and therefore provides a greater national and international scope for continuing education in oral cancer, which should be evidenced by an increase in early detection to improve survival, in this case in low- to middle-income Latin American countries [19].

Ensuring that professionals are informed about the most cost-effective strategies, such as oral self-examination, in addition to a thorough dental examination, increases early diagnosis and reduces the economic burden related to the treatment of advanced diseases. Other strategies, such as screening in risk groups, are expensive since they must be continuous and require the training of professionals for the identification of OPMD [20]. These strategies are especially valuable in countries where incidences are relatively high; others should implement the education of professionals in private practice so that, through careful examination of their patients, they can contribute to early diagnosis, in addition to being linked to the public health system and registry of the country, as an opportunity for developing countries, where resources are scarce [21].

Health professionals must know the detailed sociodemographic conditions of their patients, keep detailed and complete medical records, provide educational guidance to manage habits, promote periodic dental visits and vaccinations, and improve diet to reduce risk. As reported by Saleh et al. [22], other studies have successfully implemented the ISAC protocol using interventions to improve (I) patient information on routine oral cancer screening; screen (S) according to clinical guidelines; advise (A) high-risk patients on how to conduct a self-examination; and connect (C) patients with specialized services if necessary. This methodology was applied in the study by Jafer et al. [23], which showed a notable effect for the early diagnosis of oral cancer. Importantly, the sustainability of educational interventions, including MOOCs, requires their incorporation into continuing health education programs and, in the long term, public health policies. In addition to preserving the knowledge acquired, these strategies promote the habit of learning skills, such as oral self-examination and the early identification of potentially malignant disorders [24].

Finally, the educational strategy designed as a MOOC intervention generated higher scores for knowledge, attitudes, and practices as well as a larger effect size than did the brochure strategy. It is hoped that in the long term, the effects of this intervention can be evaluated.

## Data Availability

Information available upon request via e-mail gloria.aranzazu@ustabuca.edu.co.

## Code Availability

De-identifed data will be provided upon request.
